# Preparedness of pre-intern medical graduates of three universities in Sri Lanka to diagnose and manage anaphylaxis

**DOI:** 10.1186/s12909-021-02588-w

**Published:** 2021-03-09

**Authors:** Chandrani Nirmala Wijekoon, Indika Wettasinghe, Dinithi Fernando, Arosha Sampath Dissanayake, Malinda Gunawardana, Gayani Minuwanpitiya, Palinda Thenuwara

**Affiliations:** 1 Department of Pharmacology, Faculty of Medical Sciences, University of Sri Jayewardenepura, Gangodawila, Nugegoda, Sri Lanka; 2 Department of Physiology, Faculty of Medicine, University of Colombo, 25, Kynsey Road, Colombo 08, Sri Lanka; 3 Department of Medicine, Faculty of Medicine, University of Ruhuna, PO Box 70, Galle, Sri Lanka

**Keywords:** Anaphylaxis, Diagnosis, Management, Knowledge, Self-confidence, Pre-intern medical graduates, Sri Lanka

## Abstract

**Background:**

Early recognition and the optimal management of anaphylaxis saves lives but studies from different countries have demonstrated gaps in knowledge and practices between healthcare workers. There is a paucity of such data from Sri Lanka. We assessed knowledge, perception and self-confidence in the diagnosis and management of anaphylaxis amongst pre-intern medical graduates who would soon become first-contact doctors attending emergencies.

**Methods:**

This cross-sectional study included pre-interns who graduated with Bachelor of Medicine, Bachelor of Surgery (MBBS) degrees in 2019 from three Sri Lankan universities with differing undergraduate curricula. Using consecutive sampling data were collected within four months of the final-MBBS examinations with a self-administered questionnaire and the answers on case diagnosis and management were used as the basis of outcome scores.

**Results:**

385 participants responded (response rate: 91.5%). 16.4% correctly identified all anaphylaxis triggers. Only 7.3% correctly diagnosed all ten case scenarios and 34.5% all seven cases of anaphylaxis. 98.2 and 97.9% correctly identified 1:1000 adrenaline as the first-line treatment and the intramuscular route. 9.9% would preferentially but incorrectly use the intravenous route if access was available. Only 79.2 and 55.6% knew the correct adult and paediatric doses of adrenaline and 50% agreed that follow-up care was needed. The mean scores for case diagnosis and management of anaphylaxis were 7.7/10 ± 1.4 and 16.9/20 ± 1.9, respectively. Multiple linear regression indicated that the final MBBS results classification (class of degree or no class indicated) was a positive predictor of case diagnosis score [class vs no class: B = 0.662 (95% CI 0.347–0.978), *p* < 0.001] and being a graduate of University 2 [B = 1.568 (95% CI 1.182–1.953), p < 0.001] and passing with a class at final MBBS [B = 0.716 (95% CI 0.319–1.113), p < 0.001] were positive predictors of management score. Self confidence in diagnosing and managing anaphylaxis were rated as 79.7 and 62.1% and there was a positive correlation between knowledge and perception scores and self-confidence (case-diagnosis: *r*_pb_ = 0.111, *p* = 0.03; management: *r*_pb_ = 0.164, *p* = 0.001).

**Conclusions:**

Knowledge, perception and self confidence in the diagnosis and management of anaphylaxis was sub optimal amongst pre-interns and we identified areas that need improvement. A higher MBBS qualification classification was a predictor for correct diagnosis and management and confidence in diagnosis and management positively correlated with knowledge and perception scores. Further and enhanced educational and training strategies are needed for this life threatening emergency condition.

**Supplementary Information:**

The online version contains supplementary material available at 10.1186/s12909-021-02588-w.

## Background

Anaphylaxis is a life-threatening, acute hypersensitivity reaction affecting multiple organ systems including the cardiovascular, respiratory, gastrointestinal systems and skin [1–5]. It occurs within minutes or hours following exposure to an allergen. It progresses rapidly and usually reaches its peak within 5–30 min. Protracted anaphylaxis, a rare manifestation, can last several days. The most frequent aetiological factors include food items, medications and insect stings [1, 4]. However, anaphylaxis can occur following exposure to any substance with the ability to set off systemic degranulation of mast cells and basophils and can thus occur in any setting. Prompt diagnosis and optimal management is of utmost importance in reducing morbidity and mortality and adequate knowledge and skills in diagnosing and managing anaphylaxis is thus a pre-requisite.

As the incidence of allergies is increasing, anaphylaxis is encountered more frequently. An increasing trend has been reported in children and young adults [6–8]. The incidence shows a wide variation, with systematic reviews reporting a range from 1 to 761 per 100,000 person-years in children [8] and 1.5 to 7.9 in the general population [9]. There are no studies on the incidence or prevalence in Sri Lanka.

In the emergency setting the diagnosis of anaphylaxis is based on the clinical presentation and the key is pattern recognition. The onset is acute, usually within 2 h of allergen exposure, with rapid progression. Typically, clinical features involve two or more systems including skin and mucous membranes, cardiovascular, respiratory and gastrointestinal systems [1, 2, 4]. The involvement of more than one system is characteristic and helps to differentiate life-threatening anaphylaxis from simple allergy, which lies at the opposite end of the spectrum of hypersensitivity reactions. However, in the context of hypotension after exposure to a known allergen in a particular patient, anaphylaxis is diagnosed with systemic involvement of a single body system [1].

There are set criteria for diagnosing anaphylaxis across different contexts. These are described in guidelines developed by the World Allergy Organization (WAO), the American Academy of Allergy, Asthma and Immunology (AAAAI), the American College of Allergy, Asthma and Immunology (ACAAI), and the European Academy of Allergy and Clinical Immunology (EAACI) [1, 2, 4, 5]. These criteria are highly sensitive (96.7%) and specific (82.4%) [4].

The first-line treatment in anaphylaxis is adrenaline, a life saving drug. Adrenaline is administered intramuscularly to the mid-anterolateral thigh, in a dose of 0.01 mg/kg of a 1:1000 (1 mg/mL), up to a maximum of 0.5 mg in adults and 0.3 mg in children as soon as anaphylaxis is diagnosed or strongly suspected [1–5]. Glucocorticoids, antihistamines and nebulisation with beta-2 adrenergic agonists comprise second-line therapy [1] and have a supportive role. The immediate life-threatening manifestations of anaphylaxis, such as hypotension and angioedema, are relieved only by adrenaline.

Although mortality and morbidity due to anaphylaxis can be minimized with prompt diagnosis and appropriate management, studies from developed and developing countries have shown that its management is sub-optimal [10, 11]. In Sri Lanka, clinical experience from deaths due to anaphylaxis suggests that it continues to be managed sub-optimally.

Studies from different countries indicate that there are gaps in knowledge, attitudes and practices about anaphylaxis amongst health care workers [12–18]. The only previous study from Sri Lanka found that there were multiple deficiencies in knowledge and practice among first contact doctors in one district [19].

The aim of our study was to assess knowledge, perception and self-confidence around the diagnosis and management of anaphylaxis in recently qualified medical graduates awaiting their internship appointments. They would soon become the first contact doctors primarily responsible for dealing with anaphylaxis in hospital settings. The study was expected to identify potential deficiencies in their diagnosis and management abilities and to facilitate improvements in undergraduate and professional development programmes.

Most previous studies have included practicing doctors but none have evaluated pre-interns [12–19]. A study from India included medical and nursing students and interns [14] and most have concentrated on the emergency management of anaphylaxis [12–14, 16, 17]. Apart from a study from Sri Lanka none have evaluated knowledge around triggers [12–19]. Diagnostic skills were assessed using case scenarios in two studies [18, 19] but long term management is a further area which has not been evaluated in most studies. No previous study has assessed levels of self-confidence in the diagnosis and management of anaphylaxis. Our study, on pre-interns, included a combination of salient factors including potential anaphylaxis triggers, knowledge of diagnosis, awareness of acute and long-term management requirements and levels of self-confidence in diagnosing and management.

## Methods

The study was conducted adhering to relevant guidelines and regulations.

### Study setting and participants

This was a cross-sectional study which included pre-interns who graduated MBBS in 2019 from three selected Sri Lankan universities with different curricula. The four pre-interns who collected data were excluded. In Sri Lanka there are eleven state universities that offer undergraduate medical education and these three were selected purposively, based on feasibility. Their graduates represent a third of all annual medical graduates and the three chief types of medical curricula used in Sri Lankan medical schools. Two of the study universities, Universities 1 and 2, are located in the Western Province and University 3 in the Southern Province.

University 1 has a system-based curriculum with horizontal and vertical integration spanning three phases: preclinical, paraclinical and clinical. Although their teaching is system-based, the assessments are subject-based. In University 2, the curriculum is system-based and organized into five streams: Basic Sciences, Applied Sciences, Community, Behavioural Sciences and Clinical Sciences. The Basic Sciences stream is in the pre-clinical phase and the Applied Sciences stream is in the para-clinical phase. In the Applied Sciences Stream the assessments are system-based whereas in the other streams they are subject-based. The entire curriculum of University 3 is subject-based. The teaching and assessments are both conducted in a subject-based manner in the preclinical, paraclinical and the clinical phases. In all three universities clinical training starts in the third year and the students receive full time clinical training in the final year. The final MBBS assessments of the three universities, comprising theory and clinical examinations, are comparable. The Multiple Choice Questionnaire (MCQ) component of the final year subjects is held as a common examination for all the faculties of Medicine in the country. In the three universities, the paraclinical phase has dedicated teaching slots on anaphylaxis. During the clinical training there is no dedicated time allocated to teaching anaphylaxis, and the learning is opportunistic depending on the availability of such patients. Paraclinical and clinical assessments include questions on anaphylaxis.

### Sampling

Consecutive sampling was done using the final MBBS examination result sheets of the three medical faculties as the sampling frame. All who successfully graduated in 2019 were invited to join the study. The intended sample size was 421 (University 1, 146; University 2, 184; University 3, 91).

### Study instruments

The study instrument was a questionnaire of two parts (Additional file 1). Part A was self-administered and obtained demographic and background details such as age, gender, personal and family history of allergy and anaphylaxis to assess perceptions and self-confidence in diagnosis and management. The questionnaire comprised case scenarios, true/false questions, single answer MCQs and open-ended questions. Of the ten case-scenarios in Part A (section 2.1–2.10), the most likely diagnosis was anaphylaxis in seven, allergy in two and “other” in one. The correct responses to the questions were based on the 2011 World Allergy Organization Guidelines for the Assessment and Management of Anaphylaxis [1]. Part B of the questionnaire recorded the categorization of the final MBBS results (class of degree) from the official result sheets. Face validity of the self-administered questionnaire was assessed by two experts including a clinician.

### Data collection procedure

The contact details of all those who were in the sampling frame were recorded and they were contacted via email or telephone. When a potential participant agreed to receive the electronic versions of the study documents (on Google forms), an email with a unique study number and a link to the Google form was sent to them. The form consisted of the participant information sheet, the consent form and Part A. Clarifications related to study participation were provided when requested. The data collected from Part A was linked with degree classification data from Part B. The research team did not have access to raw marks for any of the respondents. The degree classifications were categorized into four: first-class, second class upper division, second class lower division and pass. Data from the forms were incorporated into a password protected SPSS spreadsheet and data cleaning performed by the principal investigator. Data collection was done in 2019, from University 1 in April; 2 in August and 3 in September. No incentives were provided for participation.

### Statistical analyses

Data were analysed using SPSS 21. Percentages and means were used to describe the characteristics of the study population and performance with regard to knowledge, perception and self-confidence. In the self-administered questionnaire, the 10 case scenarios for the diagnosis of anaphylaxis and the 20 questions related to the management, were equally weighted. Every correct answer scored one point and incomplete or incorrect responses scored zero. The Chi squared test, independent sample t-test and one-way ANOVA were used to assess the statistical significance of associations. The Tukey test was performed post-hoc for ANOVA. Multiple linear regression analysis was used to describe the predictors of scores for diagnosis and management. The correlation between the self-confidence and scores for knowledge and perception was assessed with point biserial correlation and *p* values less than 0.05 were considered to be statistically significant.

### Ethical considerations

The Ethics Review Committee of the Faculty of Medical Sciences, University of Sri Jayewardenepura, Sri Lanka approved the study [Ref. No.03/19]. Study participants provided informed consent prior to recruitment and their contact details, required for communication purposes, were collected and stored separately and not included in the SPSS data sheet.

## Results

Of 421 eligible subjects, 385 responded, providing a response rate of 91.5% (University 1, *n* = 135, response rate 92.5%; University 2, *n* = 170, response rate 92.4%; University 3, *n* = 80, response rate 87.9%). The mean age was 27.3 ± 1 years (range23–29 years) and 64.2% were women. The mean duration from the final MBBS examination to the time of data collection was 2.7 ± 1 months. The response rate and the characteristics of the study participants from the three universities are detailed in Table 1.

**Table 1:** Response rate and the characteristics of the study participants (*n* = 385)

	Overall (***N*** = 385)	University 1 (***N*** = 135)	University 2 (***N*** = 170)	University 3 (***N*** = 80)
**Response rate**	91.5% (385/421)	92.5% (135/146)	92.4% (170/184)	87.9% (80/91)
**Mean duration from final MBBS to data collection (months)**	2.7 ± 1	4 ± 0	2 ± 0	2 ± 0
**Mean age (years)**	27.3 ± 1	27.0 ± 1	27.3 ± 1	27.5 ± 1
**Women (%)**	64.2	60.7	60.6	77.5
**Results category (%)**				
**First-class**	4.9	6.7	5.3	1.3
**Second-class upper division**	26.0	32.6	30.0	6.3
**Second-class lower division**	40.5	37.0	44.1	38.8
**Pass**	28.6	23.7	20.6	53.8
**Personal history of allergy present (%)**	31.7	23.7	40.6	26.3
**Family history of allergy present (%)**	35.3	27.4	38.2	42.5

### Knowledge regarding triggers of anaphylaxis

Only 16.4% of the respondents correctly identified all the 33 triggers provided. Even though anaphylaxis triggered by inhalant allergens is rare, 80.8% selected each of pollen and animal fur as triggers. More than 80% identified common triggers such as shellfish, fish, beef, pork, cow’s milk, egg, pineapple, tomato, peanuts, tree nuts, penicillins, cephalosporins, quinolones, NSAIDs, blood and blood products, contrast media, vaccines, anti-venom serum, anti-rabies serum, latex and cosmetic products. However, locally relevant triggers such as chickpeas (Kadala), green gram (Mung), green leaves like spinach and Sarana (*Boerhavia diffusa)*, coconut, banana and rice were correctly identified by ≤40%. Wheat, sesame and soy were identified as triggers by 55.3, 56.4 and 63.1%. Based on university, there were differences observed in knowledge regarding certain triggers (Additional File 2 – Supplementary Table 1).

### Knowledge and perception regarding diagnosis of anaphylaxis

**Fig. 1:**
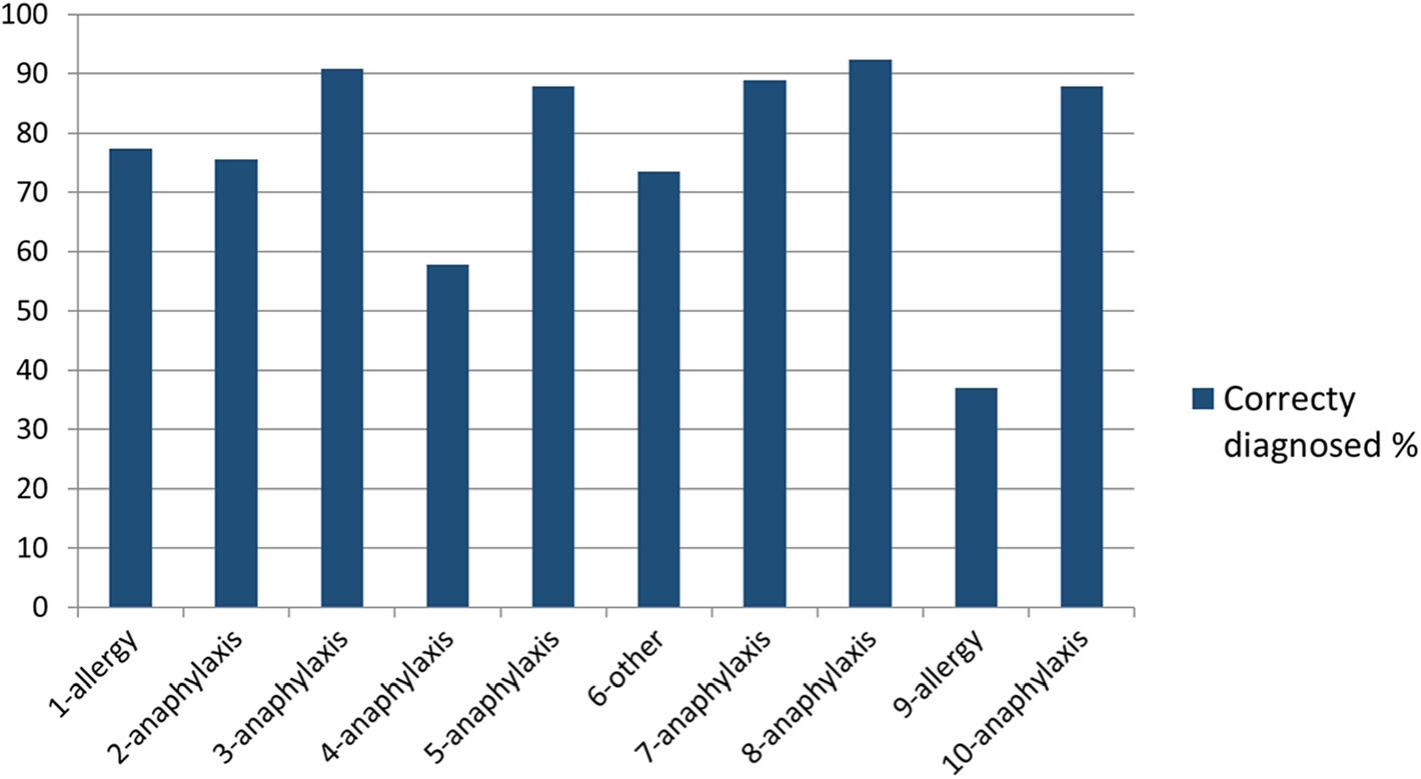
Diagnosis of case scenarios

Only 7.3% correctly diagnosed all 10 case scenarios provided. Out of the 10 cases, seven were anaphylaxis and 34.5% diagnosed all correctly. Universities 2 and 3 did better than University 1 with regard to the diagnosis in cases of anaphylaxis (*p* = 0.03). (Additional File 2 – Supplementary Table 2). Overall, the worst performance was with regard to Case 9 where the correct diagnosis was allergy [case scenario: A patient with a history of allergy to ibuprofen develops acute onset wheezing with no other symptoms or signs after taking diclofenac]. Only 36.9% diagnosed it correctly as allergy and 49.1% thought it was anaphylaxis. The second worst performance was with regard to Case 4 where the correct diagnosis was anaphylaxis [case scenario: A patient with history of allergy to prawns, develops vomiting, diarrhoea and hypotension an hour after eating “Sarana” (a green leafy vegetable)]. 57.9% diagnosed this as anaphylaxis and 34.8% thought the diagnosis was something else. (Fig. 1). 10.7 and 6.3% were unaware that anaphylaxis can occur without hypotension and skin manifestations and 16% did not know that criteria for diagnosing anaphylaxis existed. (Additional File 2 – Supplementary Table 2).

### Submitted separately

The overall mean score for case diagnosis was 7.7 ± 1.4 out of 10. The Shapiro-Wilks test (all *p* values < 0.05) and visual inspection of the histograms, normal Q-Q plots and box plots indicated that the case diagnosis score showed linearity in different categories related to gender, university, final MBBS results, and personal and family history of allergy. There was a significant difference in the mean score for case diagnosis based on the final MBBS results category (*p* < 0.001) (Table 2). Post-hoc analysis with the Tukey test showed that there was a significant difference between first class vs second lower, first class vs pass without a class, second upper vs pass without a class and second lower vs pass without a class (all p values < 0.05). Multiple linear regression analysis showed that results indicating high performance at the final MBBS positively predicted the case diagnosis score [first class vs no first class: B = 0.887 (95% CI 0.261–1.514), *p* = 0.006; class vs no class: B = 0.662 (95% CI 0.347–0.978), p < 0.001] (Table 3).

**Table 2:** Mean score for case-diagnosis and management of anaphylaxis (*N* = 385)

	Mean score for case-diagnosis (out of 10)	***P*** value	Mean score for management (out of 20)	***P*** value
**Overall**	**7.72 ± 1.4**		**16.98 ± 1.9**	
University: 1	7.59 ± 1.5	0.350^#^	16.36 ± 1.7	< 0.001^#†^
2	7.78 ± 1.3		17.96 ± 1.5	
3	7.83 ± 1.2		15.96 ± 2.1	
Final MBBS results category:		< 0.001^#†^		< 0.001^#†^
First class	8.68 ± 0.8		17.97 ± 1.5	
Second upper	7.91 ± 1.3		17.24 ± 1.8	
Second lower	7.81 ± 1.4		17.22 ± 1.8	
Pass	7.25 ± 1.3		16.23 ± 2.0	
Gender: Men	7.57 ± 1.5	0.095^##^	17.04 ± 1.9	0.699^##^
Women	7.81 ± 1.3		16.96 ± 1.9	
Personal history of allergy: Yes	7.78 ± 1.6	0.584^##^	17.27 ± 2.0	0.049^##†^
No	7.70 ± 1.3		16.85 ± 1.9	
Family history of allergy: Yes	7.82 ± 1.4	0.323^##^	17.15 ± 1.9	0.199^##^
No	7.67 ± 1.4		16.89 ± 1.9	

^#^*p* value based on one-way ANOVA; ^##^*p* value based on independent sample t test; ^†^*p* value < 0.05

**Table 3:** Simple and multiple linear regression for case-diagnosis score with participant characteristics (*N* = 385)

Model		Unstandardized Coefficients		Standardized Coefficients	t	Sig.	95% Confidence Interval for B	
		B^a^	Std. Error	Beta			Lower Bound	Upper Bound
**Simple linear regression**								
University 1 vs other universities		−0.211	0.147	−0.073	−1.434	0.152	− 0.500	0.078
University 3 vs other universities		0.130	0.173	0.038	0.750	0.454	−0.211	0.470
first class vs no first class		1.012	0.321	0.159	3.157	0.002^†^	0.382	1.642
class vs no class		0.647	0.153	0.210	4.203	0.000^†^	0.342	0.944
Personal history of allergy		0.083	0.151	0.028	0.548	0.584	−0.214	0.380
Family history of allergy		0.145	0.147	0.051	0.990	0.323	−0.143	0.434
Gender		−0.244	0.146	−0.085	−1.673	0.095	−0.532	0.043
**Multiple linear regression**								
1	(Constant)	7.269	.190		38.290	.000	6.896	7.642
	University 1 vs other universities	−.179	.156	−.062	−1.146	.252	−.486	.128
	University 3 vs other universities	.276	.193	.081	1.435	.152	−.102	.655
	first class vs no first class	.887	.319	.140	2.784	.006^†^	.261	1.514
	class vs no class	.662	.161	.216	4.125	.000^†^	.347	.978
	Personal history of allergy	.012	.158	.004	.074	.941	−.300	.323
	Family history of allergy	.002	.156	.001	.016	.987	−.304	.309
	Gender	−.185	.146	−.065	−1.267	.206	−.473	.102

^a^Multiple Linear Regression used Enter Method; ^†^*p* value < 0.05

### Knowledge and perception regarding management of anaphylaxis

98.2% of the respondents correctly identified 1:1000 adrenaline as the first-line treatment and 97.9% selected the intramuscular route. However, 13% incorrectly thought that salbutamol nebulisation was the first-line treatment if wheezing was the prominent symptom, whereas 9.9% were of the impression that if there is access, adrenaline must be administered intravenously. Only 79.2 and 55.6% stated the correct adult and paediatric doses of 1:1000 adrenaline. Ischaemic heart disease, a history of hypertension, tachycardia and pregnancy were considered contraindications for adrenaline by 7.8, 4.7, 6.5 and 1.9%, respectively. More than 20% had deficiencies related to knowledge about site of administration of intramuscular adrenaline, the positioning of the patient and intravenous fluid management. 26.8% thought that a person with a past history of anaphylaxis should never be future exposed to any substance identified as allergens in humans. 50% did not know that follow up care was needed those who have had anaphylaxis but 96% were aware of adrenaline self-use auto-injectors. University 2 responders did better regarding questions on the emergency management. (Additional File 2 – Supplementary Table 2).

The mean score for the management of anaphylaxis was 16.9 ± 1.9 out of 20. The Shapiro-Wilks test (all *p* values < 0.05), visual inspection of the histograms, normal Q-Q plots and box plots indicated that the management score showed linearity in different categories related to gender, university, final MBBS categorisation, personal and family history of allergy. There was a significant difference in the mean score for management based on the final MBBS results category (*p* < 0.001), the university (p < 0.001) and the presence of a personal history of allergy (*p* = 0.049) (Table 2). Post-hoc analysis with the Tukey test showed that there was a significant difference between first class vs pass without a class, second upper vs pass without a class and second lower vs pass without a class (all *p* values < 0.05). Post hoc analysis also showed that the management score significantly differed between University 1 and University 2 and between University 2 and University 3 (p values < 0.001 in both). On multiple linear regression analysis, being a graduate of University 2 [B = 1.568 (95% CI 1.182–1.953), *p* < 0.001] and having a class at final MBBS [B = 0.716 (95% CI 0.319–1.113), p < 0.001] were positive predictors of higher mean scores for the management of anaphylaxis (Table 4).

**Table 4:** Simple and multiple linear regression for score for management of anaphylaxis, with participant characteristics (*N* = 385)

Model		Unstandardized Coefficients		Standardized Coefficients	t	Sig.	95% Confidence Interval for B	
		B^a^	Std. Error	Beta			Lower Bound	Upper Bound
**Simple linear regression**								
University 2 vs other universities		1.755	0.176	0.455	9.992	0.000^†^	1.410	2.100
University 3 vs other universities		−1.298	0.232	−0.275	−5.592	0.000^†^	−1.754	− 0.841
First class vs no first class		1.041	0.449	0.118	2.319	0.021^†^	0.158	1.924
Class vs no class		1.117	0.210	0.262	5.310	0.000^†^	0.703	1.531
Personal history of allergy		0.413	0.209	0.100	1.973	0.049^†^	0.001	0.825
Family history of allergy		0.263	0.204	0.066	1.288	0.199	−0.139	0.665
Gender		0.079	0.204	0.020	0.387	0.699	−0.322	0.480
**Multiple linear regression**								
1	(Constant)	15.729	.233		67.646	.000	15.272	16.187
	University 2 vs other universities	1.568	.196	.406	7.987	.000^†^	1.182	1.953
	University 3 vs other universities	−.167	.248	−.035	−.673	.501	−.656	.321
	First class vs no first class	.736	.401	.083	1.838	.067	−.051	1.524
	Class vs no class	.716	.202	.168	3.546	.000^†^	.319	1.113
	Personal history of allergy	.025	.199	.006	.126	.900	−.367	.417
	Family history of allergy	.115	.196	.029	.587	.557	−.270	.500
	Gender	−.007	.184	−.002	−.037	.971	−.368	.355

^a^Multiple Linear Regression used Enter Method; ^†^*p* value < 0.05

### Source of knowledge and skills

50.9% of respondents claimed that they knew about anaphylaxis before entering the university and 99 and 93.8% respectively agreed that they gained knowledge and skills related to anaphylaxis in their medical faculty. Overall, the most frequently reported source of knowledge during undergraduate years was teaching in the para clinical phase (83.6%) and the most frequently reported source of skills were the final year clinical appointments (67.5%). There were differences based on the university (Additional File 2 – Supplementary Table 3). A large proportion of graduates from Universities 2 and 3 reported that after qualifying with MBBS, they had attended workshops and seminars where they had gained knowledge and skills in the diagnosis and management of anaphylaxis.

### Self-confidence in diagnosing and managing anaphylaxis

Overall, 79.7% were self-confident in diagnosing but only 62.1% were self-confident in managing anaphylaxis. The main reason given for lack of self-confidence in managing anaphylaxis was not having had sufficient hands-on experience. Significant differences in self-confidence levels were observed between universities (Table 5). There was a positive correlation between knowledge and perception scores and the presence of self-confidence (case-diagnosis score and self-confidence in diagnosis: *r*_pb_ = 0.111, *p* = 0.03; score for management and self-confidence in management of anaphylaxis: *r*_pb_ = 0.164, *p* = 0.001).

**Table 5:** Self-confidence in diagnosis and management of anaphylaxis (*N* = 385)

	Overall (***N*** = 385)	University 1 (***N*** = 135)	University 2 (***N*** = 170)	University 3 (***N*** = 80)	***P*** value^#^
**I am confident in diagnosing anaphylaxis (%)**	79.7	71.1	84.7	83.8	0.008^†^
**I am confident in managing anaphylaxis (%)**	62.1	52.6	75.9	48.8	< 0.001^†^
**If you are not confident in managing anaphylaxis, what are your concerns? (%)**					
I have never seen emergency management of patients with anaphylaxis	5.4	5.2	7.0	2.4	0.329
I have seen emergency management of patients withanaphylaxis but I do not have hands-on experience	32.5	42.2	17.1	48.8	< 0.001^†^
I am scared about the adverse effects of the drugs used	0.5	1.5	0	0	0.155

^#^*p* value based on Chi-square test; ^†^*p* value < 0.05

## Discussion

A pre-intern medical graduate will have undergone 5 years of medical education, acquired their MBBS degree and is awaiting the one year of compulsory internship in a service setting prior to full registration as a medical practitioner. Subsequent to acquiring the MBBS degree, they have an interim few months until the internship starts. The participants for the study were drawn from this group of ‘doctors’.

The principal findings of the study provide an overview of these pre-intern doctors’ competence and areas for improvement in the diagnosis and management of anaphylaxis. Case scenarios were used to assess their knowledge and perceptions and their ability to identify non-anaphylactic allergic reactions. Less than 10% could correctly differentiate between anaphylaxis and a simple allergic reaction in all instances. The inability to diagnose stemmed from two factors, a lack of awareness of different triggers and deficiency in knowledge on how to diagnose anaphylaxis based on criteria. Only 16% of participants could correctly identify all the recognized environmental triggers. Surprisingly, major deficiencies in knowledge were about anaphylaxis triggers specifically encountered in the local setting such as chickpeas (Kadala), green gram (Mung), green leaves like spinach and Sarana (*Boerhavia diffusa)*, coconut, banana and rice. It was notable that they were well aware of anaphylaxis triggers in the western world - this is probably the result of learning from western textbooks. 16% were unaware that anaphylaxis is diagnosed based on clinical criteria and 6% were unaware that it can occur without skin manifestations. Precise and accurate knowledge about criteria for the diagnosis and their ability to apply them was deficient.

In terms of knowledge and skills in the acute management of anaphylaxis, 98% were aware that adrenaline was the life-saving drug and that it should be given intramuscularly. Important deficiencies were noted about knowledge of adult and paediatric doses (21 and 44%, respectively) and incorrect perceptions that if access is available adrenaline administration should be intravenous (10%), that the best site of injection is the deltoid (22%), that patients should be treated in semi-recumbent position (25%), that ischaemic heart disease, pregnancy, tachycardia and hypertension are contraindications for adrenaline (2–8%) and that salbutamol nebulisation should be the first line of treatment if wheezing is the prominent symptom (13%). In relation to long term management, not arranging follow up on discharge (50%) and preventing affected persons from ever having any contact with any trigger identified as an allergen (27%) were the key deficiencies noted.

There are eleven state medical faculties in Sri Lanka and the three chosen represented different medical curriculum approaches. The pre-interns’ knowledge and skills in anaphylaxis had been acquired in different study years and in differing teaching and clinical settings. Despite these differences there was no difference in knowledge scores for diagnosis but one university (University 2) was ahead with regard to knowledge of management. The one characteristic which defined higher knowledge about diagnosis and management was overall higher academic performance. As might be expected those who qualified in a higher degree category had higher competence in anaphylaxis diagnosis and treatment. In addition to knowledge, self confidence in management was assessed as it is required to bridge the gap between knowledge and practice. Even when knowledge scores were high, only 62% had the confidence to manage a patient with anaphylaxis. The main reason quoted for this less than ideal figure was a lack of hands-on experience.

The strengths and deficiencies of the methodology of the study determine the generalisability of the findings. Although there are 11 state medical faculties, graduates of only three were included. Despite them having different student intakes our research reached out to approximately a third of the total number of all national pre-interns. As data collection was with an online questionnaire, a weakness of the study is that participants might have used external assistance to provide answers. Furthermore, although the face validity of the questionnaire was assessed further validation was not performed. The fact that the universities had different curricular models for student learning was not considered a limitation but was seen as an opportunity to assess whether any particular model of teaching was superior. Overall, our study reached 30% of all national pre-interns, had a high response rate exceeding 90% and the three chosen universities covered all the types of curricula adopted by all the medical faculties. These were strengths and support the national generalisability of the results.

In assessing how these findings compare with previous research from local and overseas settings, important differences emerge. In a previous Sri Lankan study with 98 practicing first contact doctors in the Gampaha district of Western province (the second most populous of the 25 districts in Sri Lanka), 96% knew adrenaline to be the first line treatment, 77% knew the correct route of administration, but only 31% were aware of the correct adult dose (19). Comparative figures for case scenario analysis (in the previous study there were a mix of five anaphylaxis and allergy case scenarios and in our study, there were 10), the success of doctors in previous study and pre-interns in this study were 2 and 7% respectively. These findings raise the possibility of attrition of knowledge and skills in the diagnosis and treatment of anaphylaxis over time. Similar studies from United Kingdom, United states, India, Turkey and New Zealand have reported that 45–94% had knowledge of adrenaline as the first line treatment, 9–78% knew that intramuscular route is the appropriate route and 15–33% knew the correct dose [13–18]. Comparative scores for the participants in present study were higher at 98, 98 and 79%, respectively. It should be noted that some of the studies outside Sri Lanka have been done in mixed populations of doctors, medical students, nurses and paramedics. A study from United Kingdom which included only doctors, ranging from consultants to junior doctors, reported that only 14.4% would administer adrenaline as recommended by the UK Resuscitation Council guidelines [13]. Knowledge was poor regardless of seniority or the specialty. A study from India reported that medical students performed better than interns [15]. Knowledge of diagnosis and treatment at levels similar to the present study have been reported in a study from Singapore amongst emergency department doctors. Here, 89% indicated adrenaline as the first line treatment, 85% chose intramuscular route and 73% knew the correct adult dose [19]. With regard to case scenarios, 89–94% diagnosed the three case scenarios demonstrating anaphylaxis whereas in our study there were seven case scenarios of anaphylaxis and the correct diagnosis varied from 58 to 92%. In addition, 43% of doctors incorrectly diagnosed single organ involvement without hypotension as anaphylaxis and in our study a comparable case scenario (Case 9) was diagnosed as anaphylaxis by 49%. It appears that doctors in emergency departments, quite possibly due to continuous medical education and training, retain higher levels of knowledge. Long term management aspects were studied in Turkey and 85% suggested referring the patient to an allergy clinic (15). The comparative figure from our study was 50%. In Sri Lanka there are only a few established specialised allergy clinics and this could be a reason for lack of awareness regarding follow up care.

There are several clinical and scientific implications of the findings of this study. When deficiencies in knowledge and self-confidence in the management of anaphylaxis exist, life-saving adrenaline may not reach patients at all or they may receive it in the wrong dose, resulting in severe adverse outcomes including death. Those with simple allergy, when mistakenly threated as having anaphylaxis, might also develop adverse effects unnecessarily.

The recommendation for medical educators is to formulate a structured educational programme, robust in knowledge of locally relevant anaphylaxis triggers, criteria-based diagnosis and appropriate management strategies to be implemented, along with an assessment certifying satisfactory competence. Considering that the pre-interns’ knowledge and skills were superior to that of first contact doctors in a previous study, a recommendation to medical administrators and clinical supervisors is the requirement to organise anaphylaxis related continuing medical education and in-service training to prevent de-skilling.

The study highlighted the need for future research to develop and evaluate effective educational interventions to address the deficiencies detected. Such research could identify modes of effective enhancements at undergraduate and in-service programmes. Regular clinical auditing at institutional levels may identify the optimum frequencies for ongoing refresher programmes and quality improvement initiatives need to effectively assess ongoing competency status and take urgent actions to address deficiencies.

## Conclusions

Knowledge, perceptions and self-confidence around the diagnosis and management of anaphylaxis was sub-optimal among pre-intern medical graduates in Sri Lanka. Although suboptimal, these findings from pre-interns were more favourable compared with findings from practicing doctors as in previous studies, probably because their knowledge was fresh. The study highlights the need to identify additional educational and training initiatives to narrow gaps in knowledge and practice to prepare and enhance the confidence and ability of newly graduated doctors for the provision of best care in this life-threatening emergency.

## Supplementary Informations

**Additional file 1.** Study Instrument. This is the questionnaire used for data collection.

**Additional file 2: Supplementary Table 1, Supplementary Table 2 and Supplementary Table 3.** **Supplementary Table 1.** Knowledge regarding triggers of anaphylaxis. **Supplementary Table 2.** Knowledge and perception regarding diagnosis and management of anaphylaxis. **Supplementary Table 3.**: Source of knowledge and skills related to diagnosis and management of anaphylaxis.

## Data Availability

The datasets used and/or analysed during the current study are available from the corresponding author on reasonable request.

## Abbreviations

ANOVA: Analysis of variance
FFP: Fresh Frozen Plasma
MBBS: Bachelor of Medicine, Bachelor of Surgery
NSAIDs: Non-Steroidal Anti-Inflammatory Drugs
SPSS: Statistical Package for the Social Sciences
