# Supplementary material for: Preparedness of pre-intern medical graduates of three universities in Sri Lanka to diagnose and manage anaphylaxis
Source: BMC Med Educ. 2021 Mar 9;21:152. doi: 10.1186/s12909-021-02588-w (PMC7941901; doi:10.1186/s12909-021-02588-w)
Supplement: Supplementary file 1 — Additional file 1. Study Instrument. This is the questionnaire used for data collection. [file 12909_2021_2588_MOESM1_ESM.pdf]

Additional file 1

Preparedness of pre-intern medical graduates of three universities in Sri Lanka to diagnose and manage anaphylaxis

ANNEXURE 01: Questionnaire

Pre-interns' perceptions regarding anaphylaxis

Study No: …………….

Age: ……… years Gender: Male / Female

Part A
(to be filled by the participant)

Section 1 – General

Select the most appropriate option for the statements 1.1 – 1.5

1.1 Anaphylaxis is rare in Sri Lanka	Yes / No
1.2 Anaphylaxis is a clinical diagnosis	Yes / No
1.3 There is a set of criteria to diagnose anaphylaxis	Yes / No
1.4 A house officer should be able to do the emergency management of anaphylaxis himself/herself	Yes / No

1.5 The following are known to cause anaphylaxis

fish	Yes / No	penicillins	Yes / No
shellfish (eg. prawns, lobsters, crab)	Yes / No	cephalosporins	Yes / No
beef	Yes / No	quinolones	Yes / No
pork	Yes / No	NSAIDs	Yes / No
cow's milk and dairy products	Yes / No	vaccines	Yes / No
eggs	Yes / No	blood transfusion	Yes / No
soy products	Yes / No	platelet transfusion	Yes / No
green leaves like spinach and sarana	Yes / No	FFP transfusion	Yes / No
green gram (Mung)	Yes / No	contrast media	Yes / No
chick peas (Kadala)	Yes / No	Anti-venom serum	Yes / No
coconut products	Yes / No	Anti-rabies serum	Yes / No
wheat products (bread, buns, pastries)	Yes / No	Latex gloves	Yes / No
rice/ rice flour based products	Yes / No	Plasters	Yes / No
pineapple	Yes / No	cosmetic products	Yes / No
tomato	Yes / No	animal fur	Yes / No

banana	Yes / No	pollen	Yes / No
sesame	Yes / No		
peanuts	Yes / No		
other nuts (cashew, hazelnut, almonds)	Yes / No		

Section 2 - Diagnosis of anaphylaxis

What is the most likely diagnosis for the situations from 2.1–2.10 ? Select ONE response only.

2.1 A patient with no previous history of allergy, presents with acute onset urticaria and periorbital oedema after taking diclofenac
Anaphylaxis ☐ Allergy ☐ Other diagnosis ☐

2.2 A patient with no previous history of allergy and no history of exposure to a specific allergen, presents with acute onset urticaria and wheezeing
Anaphylaxis ☐ Allergy ☐ Other diagnosis ☐

2.3 A patient with no previous history of allergy and no history of exposure to a specific allergen presents with acute onset itching, red eyes and hypotension
Anaphylaxis ☐ Allergy ☐ Other diagnosis ☐

2.4 A patient with history of allergy to prawns, develops vomiting, diarrhoea and hypotension an hour after eating “Sarana” (a green leafy vegetable)
Anaphylaxis ☐ Allergy ☐ Other diagnosis ☐

2.5 A patient with a history of allergy to tuna, accidentally eats a tuna sandwich and develops acute onset hypotension with no other symptoms / signs
Anaphylaxis ☐ Allergy ☐ Other diagnosis ☐

2.6 A patient with no previous history of allergy and no history of exposure to a specific allergen, presents with acute onset hypotension with no other symptoms / signs
Anaphylaxis ☐ Allergy ☐ Other diagnosis ☐

2.7 A patient with a history of allergy to beef, develops vomiting, abdominal pain and wheezing 15 minutes after a penicillin injection
Anaphylaxis ☐ Allergy ☐ Other diagnosis ☐

2.8 A patient with a history of allergy to pineapple, develops breathing difficulty and hypotension 30 minutes after a ceftriaxone injection
Anaphylaxis ☐ Allergy ☐ Other diagnosis ☐

2.9 A patient with a history of allergy to ibuprofen develops acute onset wheezing with no other symptoms / signs after taking diclofenac
Anaphylaxis ☐ Allergy ☐ Other diagnosis ☐

2.10 A patient with no history of allergy, presents with generalized itching and breathing difficulty following a black ant bite.
Anaphylaxis ☐ Allergy ☐ Other diagnosis ☐

Please select the best response for questions 2.11 – 2.13. Mark 'T' if you think that the statement is true and Mark 'F' if you think that the statement is false.

2.11 Anaphylaxis can occur in a patient who has no prior history of allergy	T / F
2.12 Anaphylaxis can occur without skin manifestations such as itching and urticaria.	T / F
2.13 Anaphylaxis can occur without hypotension.	T / F

<u>Section 3</u> –<u>Management of anaphylaxis</u>

3.1 What is the **FIRST** drug that need to be administered if anaphylaxis is diagnosed / strongly suspected ? (select only <u>ONE</u> response from 1-8 below)

1. Hydrocortisone
2. Chlorpheniramine
3. Promethazine
4. 1:1000 adrenaline
5. 1:10,000 adrenaline
6. Salbutamol
7. Other (specify)…………………………….
8. It depends on the symptoms and signs

3.2 Mention the adult dose of the first drug that needs to be administered in anaphylaxis.
………………………………………………………………

3.3 Mention the paediatric dose of the first drug that need to be administered in anaphylaxis.
. ……………mg/kg maximum dose : ………. mg

3.4 What is the preferred route to administer the drug you mentioned above?

1. Oral
2. Subcutaneous
3. Intramuscular
4. Intravenous
5. Nebulisation

Please select the best response for questions 3.5 – 3.20. Mark 'T' if you think the statement is true and Mark 'F' if you think that the statement is false.

3.5	Adrenaline should be given only if there is hypotension	T / F
3.6	If there is IV access in place, adrenaline must be administered intravenously	T / F
3.7	The best site to administer intramuscular adrenaline is deltoid muscle	T / F
3.8	Once given, adrenaline should not be repeated.	T / F
3.9	Adrenaline is contraindicated in patients with ischaemic heart disease	T / F
3.10	Adrenaline is contraindicated in patients with a history of hypertension	T / F
3.11	Adrenaline is contraindicated if patient presents with tachycardia	T / F
3.12	Adrenaline is contraindicated during pregnancy	T / F
3.13	If wheezing is the prominent symptom salbutamol nebulisation is the first-line treatment.	T / F
3.14	Semi-recumbent position is the most appropriate position for a patient with anaphylaxis	T / F
3.15	All patients need IV fluids	T / F

3.16 Colloids are preferred over crystalloids for IV fluid replacement T / F
3.17 On discharge, a diagnosis card needs to be issued to all patients who have had anaphylaxis T / F
3.18 On discharge, follow up care needs to be arranged for all patients who have had anaphylaxis T / F
3.19 Adrenaline auto-injectors are for self-administration of adrenaline at home T / F
3.20 A person who has developed anaphylaxis should never get exposed to any substance identified as allergens in humans T / F

<u>Section 4</u> – <u>Self-appraisal regarding anaphylaxis diagnosis and management</u>

Select the most appropriate option for the statements 4.1 – 4.5

4.1 I am confident in diagnosing anaphylaxis Yes / No
4.2 I am confident in managing anaphylaxis Yes / No
If you are <u>not</u> confident in managing anaphylaxis, what are your concerns ?
(you may select more than one option)
1. I have never seen emergency management of a patient with anaphylaxis
2. I have seen emergency management of a patient with anaphylaxis but I do not have hands-on experience
3. I am scared about the adverse effects of the drugs used
4. Other (specify)……………………………………………………………..

4.4 I recognize that I need to improve my knowledge regarding anaphylaxis Yes / No
4.5 I recognize that I need to improve my skills regarding anaphylaxis management Yes / No

<u>Section 5</u> – Source of knowledge and skills

5.1 Did you know about anaphylaxis before entering medical faculty ? Yes / No
If 'yes' what was the source of information ? (you may select more than one option)
1. School curriculum
2. Newspapers
3. Media discussions in television / radio
4. Books
5. Other (specify)…………………………………………

5.2 In the medical faculty, did you acquire knowledge related to diagnosis and management of anaphylaxis? Yes / No
If 'yes', when did you acquire such knowledge? (mark more than one if appropriate)
1. Phase I teaching (mention the exact module if you can remember) Yes / No
…………………………………………..
2. Phase II teaching Yes / No
If 'yes' specify (mark more than one if appropriate)
i. Immunology lectures/tutorials Yes / No
ii. Pharmacology lectures/tutorials Yes / No
iii. Other (specify) …………………………. Yes / No
3. 3rd year clinical appointments Yes / No
4. 4th year clinical appointments Yes / No
5. final year clinical appointments Yes / No

5.3 In the medical faculty, did you acquire skills related to diagnosis and management of anaphylaxis? Yes / No
If 'yes', when did you acquire such skills ? (mark more than one if appropriate)

1. Phase I teaching (mention the exact module if you can remember) Yes / No
   ………………………………………………..
2. Phase II teaching Yes / No
   If ‘yes’ specify (mark more than one if appropriate)
   i. Immunology lectures/tutorials Yes / No
   ii. Pharmacology lectures/tutorials Yes / No
   iii. Other (specify) Yes / No
3. 3rd year clinical appointments Yes / No
4. 4th year clinical appointments Yes / No
5. final year clinical appointments Yes / No

5.4 After qualifying with MBBS have you attended any workshops, seminars, conferences, academic meetings where you gained knowledge / skills related to anaphylaxis diagnosis and management ?

Yes/No

Section 6 – Background information

6.1 Have you got any allergies? Yes / No

If yes name the substances which are allergens for you

…………………………………………………………………………………………..

6.2 Has any of your first degree family members got any allergies? Yes / No

If yes name the substances which are allergens for him/her

…………………………………………………………………………………………..

6.3 Has any of your close friends got any allergies? Yes / No

If yes name the substances which are allergens for him/her

…………………………………………………………………………………………..

6.4 Have you ever suffered anaphylaxis ? Yes / No

6.5 Has any of your first degree family members ever suffered anaphylaxis ? Yes / No

6.6 Has any of your close friends ever suffered anaphylaxis? Yes / No

Thank you

Part B

(to be filled by the investigators)

1. **Final MBBS qualified:** Year: *2019* .. Month…………………..
2. **Final MBBS qualified attempt:** 1 / 2 / 3 / 4
3. **If qualified in the first attempt :** pass / 2nd lower class / 2nd upper class / 1st class
