# Supplementary material for: Preparedness of pre-intern medical graduates of three universities in Sri Lanka to diagnose and manage anaphylaxis
Source: BMC Med Educ. 2021 Mar 9;21:152. doi: 10.1186/s12909-021-02588-w (PMC7941901; doi:10.1186/s12909-021-02588-w)
Supplement: Supplementary file 2 — Additional file 2: Supplementary Table 1, Supplementary Table 2 and Supplementary Table 3. Supplementary Table 1. Knowledge regarding triggers of anaphylaxis. Supplementary Table 2. Knowledge and perception regarding diagnosis and management of anaphylaxis. Supplementary Table 3.: Source of knowledge and skills related to diagnosis and management of anaphylaxis. [file 12909_2021_2588_MOESM2_ESM.pdf]

Additional File 2

Preparedness of pre-intern medical graduates of three universities in Sri Lanka to diagnose and manage anaphylaxis

Supplementary Table 1: Knowledge regarding triggers of anaphylaxis (N=385)

Trigger	Overall (N=385)	University 1 (N=135)	University 2 (N=170)	University 3 (N=80)	P value[#]
Fish (yes, %)	87.3	76.3	92.9	93.8	<0.001[†]
Shellfish (yes, %)	97.4	93.3	99.4	100.0	0.001[†]
Beef (yes, %)	86.2	85.9	85.9	87.5	0.934
Pork (yes, %)	84.7	83.0	82.9	91.3	0.186
Cow's milk and dairy products (yes, %)	86.0	87.4	85.3	85.0	0.836
Egg (yes, %)	84.4	83.7	86.5	81.3	0.547
Soy products (yes, %)	63.1	57.8	64.1	70.0	0.187
Green leaves like spinach and Sarana (*Boerhavia diffusa)* (yes, %)	40.0	36.3	37.6	51.3	0.068
Green gram (Mung) (yes, %)	38.2	41.5	36.5	36.3	0.619
Chickpeas (Kadala) (yes, %)	34.3	35.6	32.9	35.0	0.882
Coconut products (yes, %)	35.1	38.5	30.6	38.8	0.262
Wheat products (yes, %)	55.3	57.0	51.8	60.0	0.419
Rice/Rice-based products (yes, %)	36.6	43.7	30.6	37.5	0.061
Pineapple (yes, %)	93.8	90.4	94.1	98.8	0.047[†]
Tomato (yes, %)	89.4	85.2	88.8	97.5	0.017[†]
Banana (yes, %)	34.0	34.8	31.2	38.8	0.485
Sesame (yes, %)	56.4	56.3	54.1	61.3	0.570
Peanuts (yes, %)	91.9	90.4	96.5	85.0	0.006[†]
Tree nuts (cashew, hazelnuts, almonds) (yes, %)	80.5	79.3	82.9	77.5	0.539
Penicillins (yes, %)	97.7	93.3	100.0	100.0	<0.001[†]

Cephalosporins (yes, %)	91.7	84.4	95.9	95.0	0.001†
Quinolones (yes, %)	89.1	88.1	87.1	95.0	0.156
NSAIDs (yes, %)	82.9	72.6	90.0	85.0	<0.001†
Vaccines (yes, %)	97.9	94.1	100.0	100.0	0.001†
Blood transfusion (yes, %)	96.4	90.4	100.0	98.8	<0.001†
Platelet transfusion (yes, %)	84.9	78.5	89.4	86.3	0.029†
FFP transfusion (yes, %)	90.1	88.9	90.0	92.5	0.690
Contrast media (yes, %)	97.7	94.1	99.4	100.0	0.003†
Anti-venom serum (yes, %)	96.9	91.1	100.0	100.0	<0.001†
Anti-rabies serum (yes, %)	96.1	93.3	97.1	98.8	0.096
Latex (yes, %)	81.3	77.8	82.4	85.0	0.378
Plasters (yes, %)	78.7	80.7	75.3	82.5	0.333
Cosmetic products (yes, %)	95.1	92.6	96.5	96.3	0.257
All Above 33 triggers (yes, %)	16.4	14.8	14.7	22.5	0.249
Pollen (yes, %)	80.8	82.2	78.2	83.8	0.511
Animal fur (yes, %)	80.8	80.0	80.6	82.5	0.901

FFP: Fresh Frozen Plasma; NSAIDs: Non-Steroidal Anti-Inflammatory Drugs

#p value based on Chi-square test; †p value < 0.05

Supplementary Table 2: Knowledge and perception regarding diagnosis and management of anaphylaxis (N=385)

Knowledge and perception regarding diagnosis of anaphylaxis

		Overall (N=385)	University 1 (N=135)	University 2 (N=170)	University 3 (N=80)	P value#
**Number of Cases correctly diagnosed (%)**	10	7.3	7.4	6.5	8.8	0.197
	9	22.3	17.8	26.5	21.3	
	8	31.9	38.5	28.2	28.8	
	7	22.9	16.3	24.7	30.0	
	6	8.1	8.1	8.2	7.5	
	5	4.9	6.7	4.1	3.8	
	4	2.1	4.4	1.2	0	
	3	0.5	0.7	0.6	0	
**All 7 cases of anaphylaxis correctly diagnosed (%)**		34.5	25.9	38.6	38.8	0.03†
**Not providing the correct answer to the following true statements (%)**						
Anaphylaxis is a clinical diagnosis		2.6	7.4	0	0	<0.001†
There is a set of criteria to diagnose anaphylaxis		16.0	17.0	14.7	16.9	0.833
Anaphylaxis can occur in a patient who has no prior history of allergy		5.5	14.8	0.6	0	<0.001†
Anaphylaxis can occur without skin manifestations		6.3	14.9	1.2	2.5	<0.001†
Anaphylaxis can occur without hypotension		10.7	19.4	4.7	8.9	<0.001†

Knowledge and perception regarding management of anaphylaxis

	Overall (N=385)	University 1 (N=135)	University 2 (N=170)	University 3 (N=80)	P value#
**First-line treatment in anaphylaxis (%)**					
1:1000 adrenaline	98.2	97.0	99.4	97.5	0.226
1:10,000 adrenaline	1.0	2.2	0	1.3	
promethazine	0.3	0	0	1.3	
it depends on symptoms and signs	0.3	0	0.6	0	

**Route od administration of first-line treatment (%)**					
intramuscular	97.9	97.8	97.6	98.8	0.715
intravenous	1.0	1.5	0.6	1.3	
subcutaneous	0.5	0.7	0.6	0	
**Adult dose correctly stated (%)**	79.2	80.0	84.1	67.5	0.01†
**paediatric dose correctly stated (%)**	55.6	35.6	78.2	41.3	<0.001†
**Not providing the correct answer to the following false statements (%)**					
Adrenaline should be given only if there is hypotension	3.1	2.2	2.4	6.3	0.193
If there is intravenous access in place, adrenaline must be administered intravenously	9.9	13.3	5.9	12.5	0.065
If wheezing is the prominent symptom, salbutamol nebulisation is the first-line treatment	13.0	17.8	7.1	17.5	0.009†
The best site to administer intramuscular adrenaline is deltoid muscle	21.6	27.4	7.6	41.3	<0.001†
Once given, adrenaline should not be repeated	1.6	1.5	1.2	2.5	0.730
Adrenaline is contraindicated in those with:					
ischaemic heart disease	7.8	9.0	4.7	12.5	0.084
history of hypertension	4.7	4.5	3.5	7.5	0.381
tachycardia	6.5	7.6	4.7	8.8	0.405
pregnancy	1.9	1.5	0	6.3	0.003†
Semi-recumbent position is the most appropriate position	25.3	36.6	12.9	32.5	<0.001†
All patients need intravenous fluids	31.7	46.7	16.5	38.8	<0.001†
Colloids are preferred over crystalloids for IV fluid replacement	6.8	11.1	4.1	5.0	0.042†
A person who has developed anaphylaxis should never get exposed to any substance identified as allergens in humans	26.8	41.5	14.7	27.5	<0.001†

**Not providing the correct answer to the following true statements (%)**					
On discharge, a diagnosis card needs to be issued for all who have had anaphylaxis	1.3	2.2	0.6	1.3	0.460
On discharge, follow up care needs to be arranged for all who have had anaphylaxis	50.0	33.6	54.7	67.5	<0.001[†]
Adrenaline auto-injectors are for self-administration of adrenaline at home	3.9	3.7	2.4	7.5	0.144

[#]p value based on Chi-square test; [†]p value < 0.05

Supplementary Table 3: Source of knowledge and skills related to diagnosis and management of anaphylaxis (N=385)

	Overall (N=385)	University 1 (N=135)	University 2 (N=170)	University 3 (N=80)	P value#
**Did you know about anaphylaxis before entering medical faculty? (yes, %)**	50.9	57.0	44.7	53.8	0.086
**In the medical faculty, did you acquire knowledge related to diagnosis and management of anaphylaxis? (yes, %)**	99.0	97.8	100.0	98.8	0.161
**If yes, when (yes, %)**					
Pre-clinical phase	32.2	33.3	34.1	26.3	0.435
Para-clinical phase	83.6	86.7	94.2	56.3	<0.001†
Para-clinical phase – immunology teaching	75.6	79.3	90.6	37.5	<0.001†
Para-clinical phase – pharmacology teaching	71.4	56.3	93.5	53.8	<0.001†
3rd year clinical appointments	77.9	74.1	76.5	87.5	0.06
4th year clinical appointments	75.3	69.6	80.5	73.8	0.088
final year clinical appointments	71.7	56.7	81.7	75.9	<0.001†
**In the medical faculty, did you acquire skills related to diagnosis and management of anaphylaxis? (yes, %)**	93.8	91.1	94.1	97.5	0.168
**If yes, when (yes, %)**					
Pre-clinical phase	9.4	14.1	8.2	3.8	0.034†
Para-clinical phase	54.0	54.8	67.1	25.0	<0.001†
Para-clinical phase – immunology teaching	45.5	45.2	59.9	16.3	<0.001†
Para-clinical phase – pharmacology teaching	44.6	35.6	60.8	26.3	<0.001†
3rd year clinical appointments	59.5	51.9	62.4	66.3	0.068
4th year clinical appointments	55.6	31.1	71.4	63.8	<0.001†
final year clinical appointments	67.5	45.9	81.8	73.8	<0.001†

**After qualifying with MBBS, have you attended any workshops, seminars, conferences etc. where you gained knowledge / skills related to diagnosis and management of anaphylaxis? (yes, %)**	46.5	5.9	67.6	70.0	**<0.001**†

#p value based on Chi-square test; †p value < 0.05
